# P23 Assessment of knowledge, attitude and practice towards antimicrobial use and resistance among students in three secondary schools in Dodoma City

**DOI:** 10.1093/jacamr/dlac004.022

**Published:** 2022-02-16

**Authors:** Erick Venant, Baritazar K. Stanley, Michael J. Mosha, Kelvin E. Msovela, DorineGrace J. Mushi, Pendo Masanja, Karin Wiedenmayer, Eva Ombaka

**Affiliations:** RBA Initiative, Dodoma, Tanzania; RBA Initiative, Dodoma, Tanzania; RBA Initiative, Dodoma, Tanzania; St John’s University of Tanzania, Dodoma, Tanzania; RBA Initiative, Dodoma, Tanzania; University of Dodoma, Tanzania; Swiss Tropical and Public Health Institute, Basel, Switzerland; University of Basel, Basel, Switzerland; St John’s University of Tanzania, Dodoma, Tanzania

## Abstract

**Introduction:**

Antimicrobial resistance is still not given enough attention and the public is insufficiently aware of its existence, leading to behaviour, which propagates the rise of antimicrobial resistance (AMR). One of the objectives of Tanzania's national action plan on antimicrobial resistance is to improve awareness and understanding of antimicrobial use and resistance through effective communication, education and training. This task will need involvement of many stakeholders and sectors.

**Objectives:**

To assess the knowledge, attitudes and practices toward antimicrobial use and resistance among students in three secondary schools in Dodoma city.

**Methods:**

For this interventional pre-post comparative study, data were collected before and after training on antimicrobial use and resistance. Secondary school students from Mkonze, Merriwa and Kiwanja cha Ndege secondary school who are members of AMR school clubs participated. Training included classroom teaching and arts and crafts. We used quantitative and qualitative data collection methods by using self-administered paper-based structured coded questionnaires delivered to the students with the supervision of school guardians. Analysis was done through Excel and SPSS.

**Results:**

Three aspects were investigated: awareness of ways to reduce AMR; knowledge that antibiotics cannot be used to treat flu and factors that contribute to AMR. Before the training knowledge of these was below 37%. Three months after the training knowledge had increased to above 90%.

**Conclusions:**

Training of secondary school students significantly improved awareness, knowledge and attitude regarding antimicrobial use and antimicrobial resistance. AMR school clubs are an effective vehicle to raise awareness and mitigate the AMR crisis. Focus on students will lead to wider awareness in the community.

**Table 1.** Shows three aspects investigated: awareness of ways to reduce AMR; knowledge that antibiotics cannot be used to treat flu and factors that contribute to AMR

Aspect investigated	Before training		After training	
	Measure	%	Measure	%
Can antibiotics be used to treat flu?	Yes	38.3	Yes	90.6
	No	50.0	No	5.6
	I don't know	11.7	I don't know	3.6
Knowledge on practices and behaviour that contribute to AMR	Aware	31.2	Aware	99.3
	Not aware	69.8	Not aware	0.7
Knowledge on the consequences of AMR	Aware	31.1	Aware	98.6
	Not aware	69.9	Not aware	1.40

